# Which community-based HIV initiatives are effective in achieving UNAIDS 90-90-90 targets? A systematic review and meta-analysis of evidence (2007-2018)

**DOI:** 10.1371/journal.pone.0219826

**Published:** 2019-07-17

**Authors:** Sailly Dave, Trevor Peter, Clare Fogarty, Nicolaos Karatzas, Nandi Belinsky, Nitika Pant Pai

**Affiliations:** 1 Division of Clinical Epidemiology, Research Institute of the McGill University Health Centre, Montreal, Canada; 2 Clinton Health Access Initiative, Gaborone, Botswana; 3 Department of Medicine, McGill University, Montreal, Quebec, Canada; British Columbia Centre for Excellence in HIV/AIDS, CANADA

## Abstract

**Background:**

Reaching the Joint United Nations Programme on HIV/AIDS (UNAIDS) 90-90-90 targets to end the HIV epidemic relies on effective interventions that engage untested HIV+ individuals and retain them in care. Evidence on community-based interventions through the lens of the targets has not yet been synthesized, reflecting a knowledge gap. We conducted a systematic review and meta-analysis to shed light on successful community-based interventions that have been effective in contributing, directly or indirectly, towards the UNAIDS 90-90-90 targets: knowledge of HIV status, linkage to care/on treatment, and viral suppression. Linkage to care was also included in this review due to the limitations of studies.

**Methods:**

We conducted a systematic review and meta-analysis of the period 2007–2018. Eleven databases were searched to identify community-based interventions designed to improve knowledge of HIV status (in particular HIV testing), linkage to care/on treatment, and/or viral suppression. Eligible studies were classified by intervention, population, country income level, outcomes and success. Success was defined as interventions demonstrating statistical significance between intervention and control group or that reached any target by proportion; 90% testing, 81% linked to care/on treatment and 73% viral suppression.

**Results:**

Of 82 eligible studies, 51.2% (42/82) reported on HIV testing (first 90), 20.7% (17/82) on linkage to care/ on treatment (second 90), and 45.1% (37/82) on viral suppression (third 90). In all, 67.1% (55/82) of studies reported success; 21 studies on the first 90, 9 towards linkage to care/on treatment, and 25 towards the third. By strategies, 36.6% deployed community workers/peers, 22% used combined test and treat strategies, 12.2% used educational methods, 8.5% used mobile testing, 7.3% used campaigns and 13.4% used technology. For HIV testing/linkage, combined test/treat interventions were often used, for viral suppression, educational interventions and technologies were commonly deployed. Our pooled analysis suggested that deployment of community health care workers/peer workers significantly improved viral suppression (pooled OR: 1.40 95% CI 1.06–1.86). Of the studies published after 2014, 50.0% reported metrics aligned with UNAIDS targets.

**Conclusions:**

Data on linkage to care/on treatment (second target) remained weak, because many studies reported successes on the first and third targets. Stratification by targets and country income levels is informative and guides adaptation of successful interventions in comparable settings. Consistent reporting of clear metrics aligned with UNAIDS targets will aid in synergy of study data with programmatic data that will help reportage. Exploration of innovative interventions, for engagement and linkage and deployment of community/ peer workers is strongly encouraged.

## Introduction

### Rationale

HIV/AIDS continues to be a public health concern worldwide; over 70 million people have been infected with HIV and close to 35 million people have died as a result since the beginning of the epidemic. As of June 2017, about 37 million were living with HIV, with about 20.9 million [18.4 million–21.7 million] accessing antiretroviral therapy [1]. According to the World Health Organization (WHO), the African region is disproportionately affected by HIV with an estimated prevalence among adults of 4.2% (3.7–4.8) [2]. In response to the growing problem, in 2014 the Joint United Nations Programme on HIV and AIDS (UNAIDS) set an ambitious target known as the 90-90-90, that indicated that by 2020, 1) 90% of all HIV positive people will be diagnosed, 2) 90% of all those diagnosed will be on treatment and 3) 90% of those linked to care will be virally suppressed. According to the UNAIDS Global AIDS Monitoring Guidelines, HIV testing is ideally measured by determining the number of people who have been diagnosed with HIV divided by the total number of people living with HIV; linkage to care is measure by dividing the number of people who are on ART by the number of people who have HIV; viral suppression is measured by the number of people achieving viral suppression divided by the number of people with HIV [3, 4]. These UNAIDS targets translate proportionally to 90% of all at risk to be tested/screened, 81% of those found positive must be on treatment and finally, 73% on treatment to be virally suppressed to stop the epidemic [5]. In order to achieve the goals by 2020, the UNAIDS 90-90-90 targets call for countries to focus on HIV screening/diagnosis, and treatment/viral suppression simultaneously [6].

According to the 2017 Global AIDS Update, many countries are making significant progress towards the 90-90-90 targets; some countries have reached all three targets, and many are close to reaching them [7]. Progress varies due to differences in local epidemics and capacity of health systems, despite WHO’s recommendation of ART initiation for all persons living with HIV [8]. A syntheses of gaps in the knowledge of HIV status, guides enrolment of the undiagnosed in care [7]. Evidence has shown there are no clear-cut methods of improving HIV testing, linkage to treatment and viral suppression. Furthermore, differing populations, country income levels, and social settings add layers of complexity when it comes to implementing interventions and determining whether they will be successful in these settings.

Countries such as Botswana and Australia, both with very different economic backgrounds have each shown that it is possible to reach some of the targets by way of concerted efforts, when resources are less readily available [7, 9]. Closing the gap requires prioritizing HIV testing and rolling out initiatives in community settings [7].

Community-based interventions have become increasingly popular, with reported decreases in HIV-related mortality, virologic failure, and other adverse health outcomes [10–13]. Decentralization of services and task-shifting to community workers in low and middle-income countries is not only cost effective, but also achieves the same results as facility based-services [14]. Furthermore, a systematic review on HIV testing in Sub-Saharan Africa demonstrated that community-based testing had higher uptake compared to facility-based testing [15, 16]. Community-based interventions are now being recognised as key to achieving the UNAIDS 90-90-90 targets through their ability to overcome structural and societal barriers in access to health care [17].

Although evidence on community-based initiatives has been synthesized prior to the announcement of the 90-90-90 targets, an updated, comprehensive review of community initiatives and outcomes, aligned to the UNAIDS targets was not available, precipitating a gap in understanding progress towards the targets [14–16, 18, 19]. Furthermore, a need for a high quality review through the lens of the UNAIDS 90-90-90 targets that could serve as an evidence base for policy makers and stakeholders playing a key role in achieving global targets was felt. With no limitation on population and with the intention of understanding various successful interventions in high, middle and low-income countries such that they could be recommended to other countries of similar income levels, we decided to undertake this review.

### Objectives

The primary objective of this systematic review and meta-analysis is to shed light on successful community-based interventions that have been effective in contributing towards the UNAIDS 90-90-90 targets, stratifying by intervention type and country income level. We also set out to determine if reporting of outcomes and/or metrics before and after the 2014 announcement of the UNAIDS targets were consistent and in line with the care continuum. With 2020 around the corner, it is vital that concise information regarding successful interventions be accessible to researchers, stakeholders and policymakers.

## Methods

The Preferred Reporting Items for Systematic Review and Meta-Analyses (PRISMA) and Cochrane guidelines were followed in reporting this review (S1 Checklist) [20].

### Protocol registration

This review is unregistered.

### Data source

We searched eight databases for the period 2007–2018; MEDLINE via PubMed, OVID, BIOSIS, LILACS, CINAHL, Cochrane DARE, Embase and Global Index Medicus, and three conferences (Conference on Retroviruses and Opportunistic Infections, International AIDS Conference and African Society for Laboratory Medicine).

### Search

The search strategy for PubMed was ("hiv"[MeSH Terms] OR "hiv"[All Fields]) OR ("acquired immunodeficiency syndrome"[MeSH Terms] OR ("acquired"[All Fields] AND "immunodeficiency"[All Fields] AND "syndrome"[All Fields]) OR "acquired immunodeficiency syndrome"[All Fields] OR "aids"[All Fields]) AND (UNAIDS[All Fields] AND/OR 90-90-90[All Fields] AND (“youth AND/OR “adults”) AND “viral load”).

### Eligibility criteria

All experimental trials and observational studies were included. In order to be included for the review, studies had to report on any or all of the UANIDS 90-90-90 targets/outcomes, that is; knowledge of HIV status by means of HIV testing, linkage to and/or retention in treatment, or virologic suppression/ART adherence. Modelling studies, as well as reviews, narratives, trial protocols and studies that were in a language other than English were excluded. Bibliographies of chosen articles were further scanned for additional articles.

### Study selection

Three reviewers (CF, NK and SD) conducted data selection and abstraction. Quality assessment was conducted by two reviewers (SD and AK).

### Data collection process

A pre-designed abstraction table was used to collect information on author, study design, study population, sample size, intervention description, HIV outcome measure, and metrics used to measure reported outcomes pertaining to 90-90-90 targets. Interventions were categorized based on country income level into high, upper middle, lower middle and low-income countries (HICs, upper MICs, lower MICs and LICs), and by type of intervention (1) Community health workers (including lay workers/peers); 2) Educational; 3) Integrated or Combined services; 4) Mobile Testing units (e.g. mobile vans); 5) Campaigns; and 6) Technology). Success was defined as reaching the proportions in either of the targets, i.e. 90%, 81%, and 73% for HIV testing, linkage to care/on treatment and viral suppression, respectfully. If statistical significance was reached between intervention and control group, it was deemed a successful intervention as well.

### Summary measures and synthesis of results for a systematic review

The outcomes that capture UNAIDS 90-90-90 targets were: 1) HIV testing, 2) Linkage to care/on treatment, and 3) Viral suppression. Pooled analysis was performed for outcomes that were consistently documented among similar interventions. A random effect meta-analysis model was used to determine pooled estimate. Forest plots were made for visual representation of heterogeneity and pooled OR with 95% CI. We performed all statistical analyses using Stata/IC, V.12.

Knowledge of HIV status was defined as any method of diagnosing/screening HIV, in particular HIV testing. Linkage to care/on treatment was defined as any way of linking recently diagnosed HIV+ patients to healthcare services and/or initiated on ART within a reasonable time from diagnosis. Linkage to care was included as a metric towards the second 90 subject to the limitations of different studies and variations in definitions. Viral suppression was defined as having viral levels <1000 copies/mL, or as defined by the individual studies themselves; in some cases ART adherence was a reported outcome, this was used as a precursor to viral suppression, the rationale being that sustained ART adherence will result in viral suppression.

Targets were reported by metrics that varied across studies, were heterogeneous and inconsistent. Metrics varied from number of HIV tests conducted, attendance rate, linkage to care, ART adherence, pill count, viral load, CD4 count and self reported outcomes. Acceptability was defined as the willingness of participants to use community-based interventions. Feasibility was defined as the convenience of implementing such interventions. The lack of consistent reporting of outcomes and/or study design precluded pooling of measures for the majority of studies and therefore pooling was only performed where possible.

### Risk of bias across studies

Quality was assessed using the Cochrane Risk of Bias Tools and the Newcastle-Ottawa quality assessment scale, for clinical trials and observational studies respectively.

## Results

### Study selection

**Fig 1:**
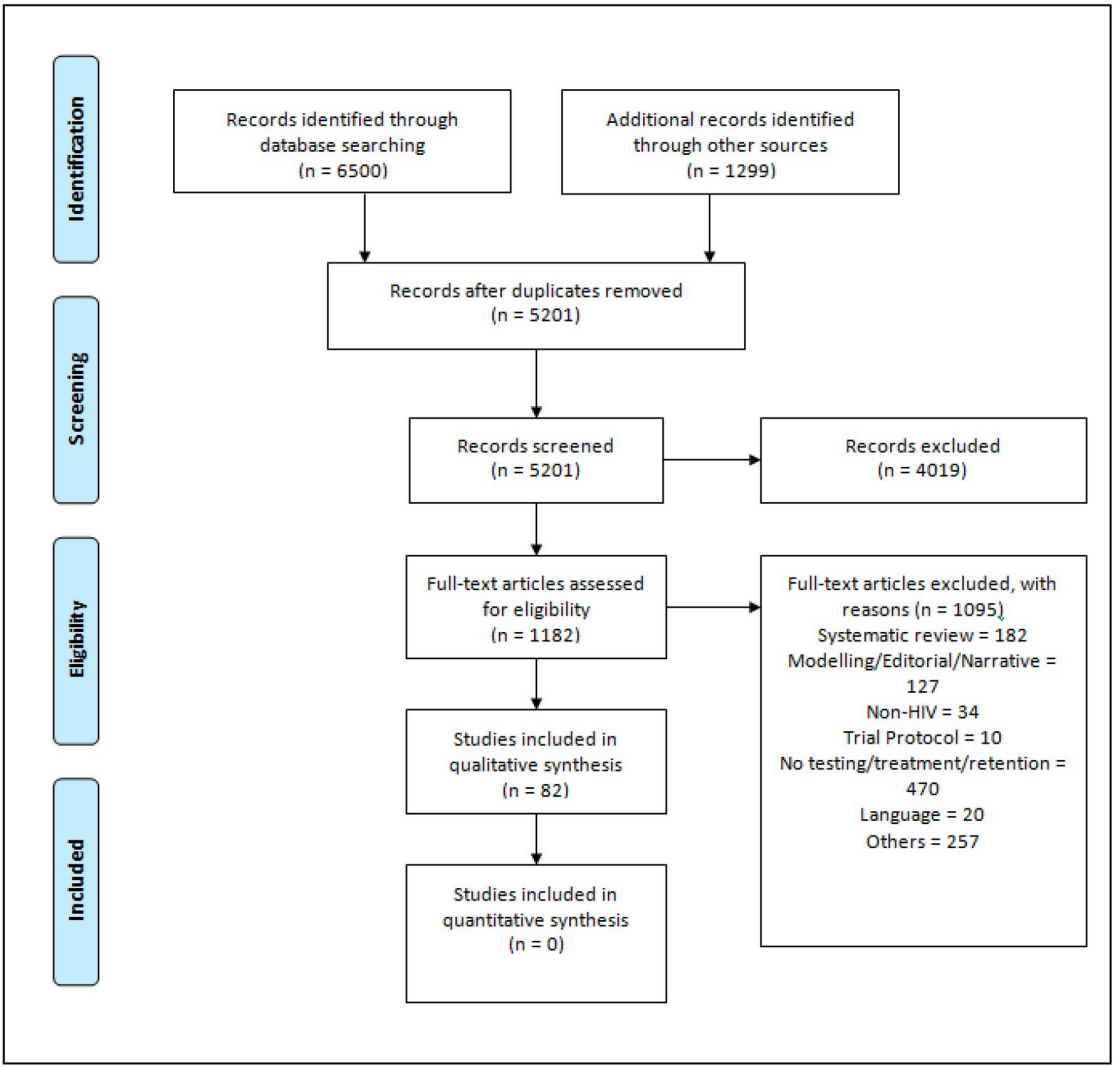
Preferred reporting items for systematic reviews and meta-analyses (PRISMA) Flow Diagram.

Over 5000 studies were identified through the search, out of which 82 studies met our eligibility criteria (Fig 1).

### Study characteristics

By WHO geographical location, 25.6% (21/82) of the studies were conducted in the Americas, 3.6% (3/82) in Europe, 3.6% (3/82) in the Western Pacific region, 6.1% (5/82) in the South East Asia region and 68.3% (56/82) in the African region. Classified by country income level, 26.8% (22/82) of the studies were conducted in high income countries, 25.6% (21/82) were from upper middle-income countries, 30.5% (25/82) were from lower middle-income countries and 24.3% (20/82) were conducted in low-income countries. Some studies had multiple locations varying by regions and country, thus contributing to percentages over 100%.

**Fig 2:**
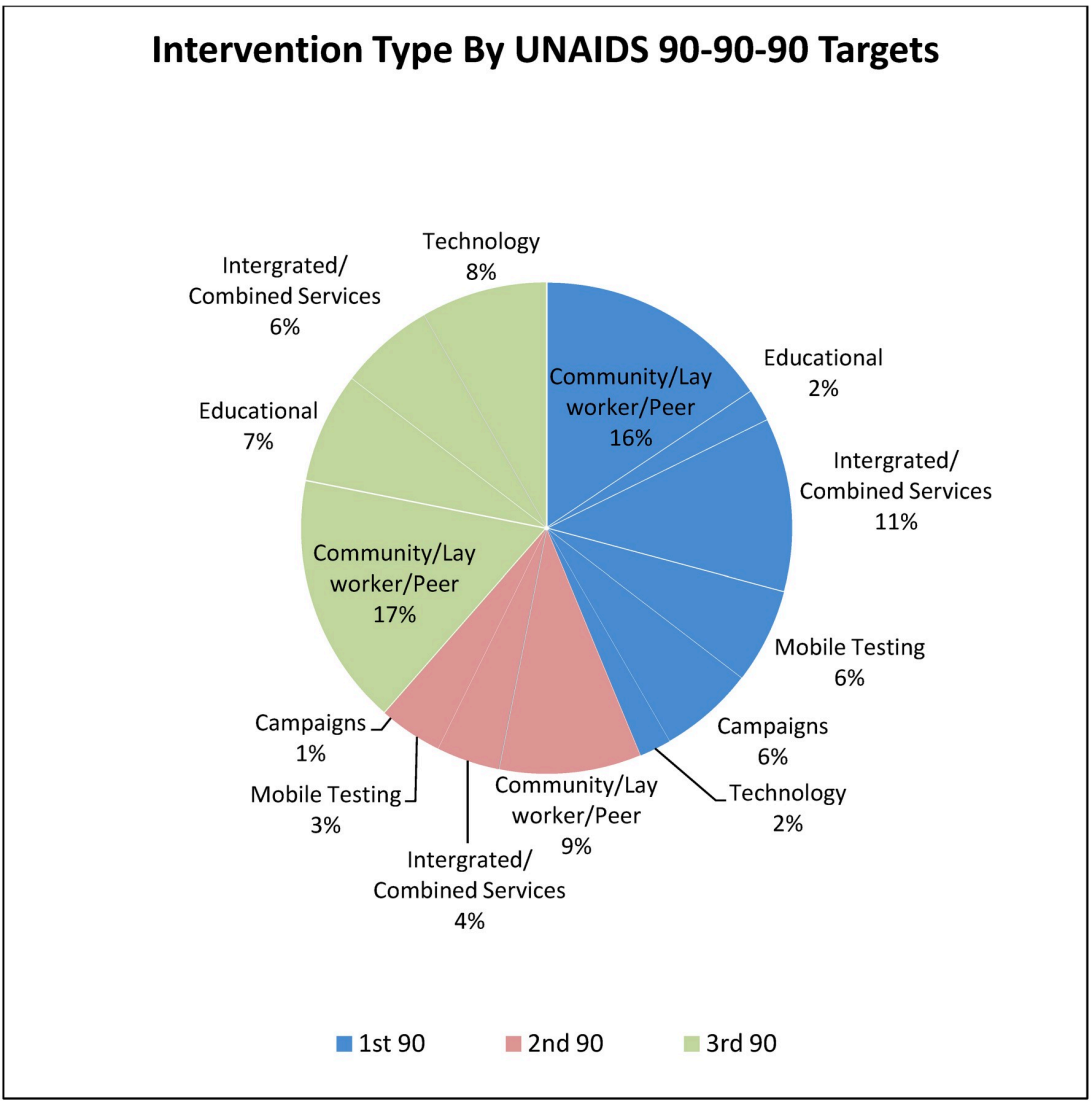
Interventions by UNAIDS 90-90-90 targets and type of intervention. Blue represents interventions related to HIV testing, pink represents interventions associated with linkage to care/treatment and green represents interventions associated with viral suppression.

Randomised controlled trials, comprised 29.3% (24/82) of the studies, followed by 24.3% (20/82) of cross-sectional studies and 14.6% (12/82) of feasibility studies. Quasi-experimental and cohort/case control studies were also noted. Overall by UNAIDS targets, a vast proportion of studies, (42), had interventions that targeted the first 90, while 17 studies targeted the second 90, and 37 studies targeted the third 90. Fig 2 shows intervention type by UNAIDS targets.

A little over half (56.1%, (46/82)) of the studies were published after the announcement of the UNAIDS targets, out of these 50.0% (23/46) reported according to metrics aligned with the UNAIDS targets. The other 50.0% reported using metrics such as self-reporting of outcomes, first-time testers, CD4 counts or other metrics not directly aligned with the indicator measures for the UNAIDS 90-90-90 targets. This is an improvement in reporting of outcomes aligned to UNAIDS targets as only 33% of studies pre-90-90-90 announcement reported outcomes according to the targets.

### Results of individual studies

A summary of all included studies can be found in the S1 Table–Summary of Study Characteristics.

#### Target 1: Knowledge of HIV status by means of HIV testing

Out of the 82 studies, 51.2% (42/82) documented HIV testing. This included any intervention that increased HIV testing uptake. Of the 42 studies, 21 (50%) were deemed successful based on statistical significance compared to a comparator group, or whether they reported proportions of testers reaching 90% (±10%) or higher based on the first UNAIDS 90-90-90 targets.

Of the 21 studies reporting positive results, 9 (42.9%) made use of link workers/community health workers, lay workers or peers to increase testing uptake. In middle income countries such as those in Southern Africa, India as well as in low income countries, successful interventions employed community health workers to conduct home-based HIV testing. In some cases couple-based HIV testing and counselling was used over individual based HIV testing and counselling [21–29]. Combination of interventions were used in 5 studies. These were particularly prominent in middle and lower income countries; deployment of such interventions included door-to-door testing, integrated HIV testing and counselling in antenatal care groups and group education. [30–34]. Four studies in South Africa and East Africa made use of mobile testing vans in rural areas. This method was particularly useful in reaching younger male populations, who are known to typically not access health facilities in this region [35–38]. Three studies involved testing campaigns; one employed network based approaches to increase screening for at-risk individuals while another testing campaign was carried out by an independent humanitarian organisation to increase testing in vulnerable populations. Finally, a behaviour change communication campaign, involving messages around testing, sexual behaviour and condom use through different channels, demonstrated significant associations between campaign exposure and HIV testing [39–41].

Five studies were acceptability or feasibility studies, of which three were highly acceptable interventions; integrated HIV testing in ANC settings, a national radio program that increased HIV knowledge and school based HIV testing and counselling [42–44]. One study was moderately acceptable, involving peer delivery of volunteering counselling and testing services [45]. Another reported on feasibility; this intervention distributed redeemable vouchers for an at-home HIV testing kit [46].

#### Target 2: Linkage to care/on treatment

Linkage to care was included and defined as those linked to HIV services (i.e., counselling, clinical care, or initiation of ART), the reason for this being that smaller scale intervention studies do not directly quantify the second 90 due to study limitations and differences in definitions.

Nine studies (52.9%) studies were deemed successful by either reaching statistical significance or reaching 81% by proportion linked to care/on treatment. Five studies used link workers/community health workers or lay workers, these were more common in middle income countries. A home-based HIV counselling and testing (HBHCT) initiative directed towards families of HIV positive patients in South Africa yielded 93.7% of those diagnosed HIV+ through this methods, another study also looking at HBHCT in South Africa demonstrated 76% were linked to care; another community-based health and social service centre in USA, provided support to youth regarding HIV and other STIs, reported 80.% of youth diagnosed with HIV met with a case manager to initiate care [25, 47, 48].

A combination of interventions such as loud speakers, flyers and campaigns, treatment in antenatal care settings and test and treat strategies [30, 49, 50] were successful in middle income countries. The test and treat strategy involved multi-disease health campaigns followed by home-based testing, streamlined ART and included patient-centered care and a viral load testing in Kenya and Tanzania proved to be successful when a multi-pronged approach was taken; one study compared linkage to care among two groups of individuals who were recruited for HIV testing via alternative venue based testing vs. social and sexual referral networks, thus demonstrating the importance of network-based testing to increase subsequent linkage to care [39].

#### Target 3: Viral suppression/ART adherence

ART adherence was defined as those adhering to ART to achieve viral suppression. Various metrics used for this target were CD4 counts, viral suppression, pill counts or self reported adherence, as these sustained behavioural changes would result in the clinical outcome of viral suppression.

Twenty five studies were deemed successful, in reaching 73% adherence/suppression by proportion, within the study population. Of the 25 studies, eleven, (44%) utilized community health workers, lay workers or other forms of people (e.g peers/family) for treatment assistance. This was particularly popular in middle and low income countries compared to high income countries. Studies conducted in South Africa, Kenya, Mozambique and Peru specifically used Directly Observed Therapy (DOT), via a community worker/lay worker, a peer or even a partner, to ensure patients took ARTs and demonstrated sustained viral suppression. Other studies used community health workers, lay workers or other forms of people (e.g peers/family) to send reminders to take medication [47, 51–59].

Five (20%) used a combination of interventions to target any or all of the cascade of care with the end goal of viral suppression; one study integrated food security alongside ART services, another provided incentives paid to community health centres on a per person basis in care, while others combined the use of adherence diaries, group education, counselling etc to ensure viral suppression. These were all studies conducted in middle or low income countries, with the exception of one conducted in China (which involved the cash on delivery model) [50, 60–63].

Four studies (16%) involved educational sessions (2 motivational counselling, 1 cognitive therapy and 1 nutritional and lifestyle educational session). All the educational-related successful interventions were conducted in middle and low income countries [64–67]; five studies used a form of technology (i.e., alarm device, text messages sent to cellphones, beepers or electronic drug monitors) [68–72].

### Pooled analysis

**Fig 3:**
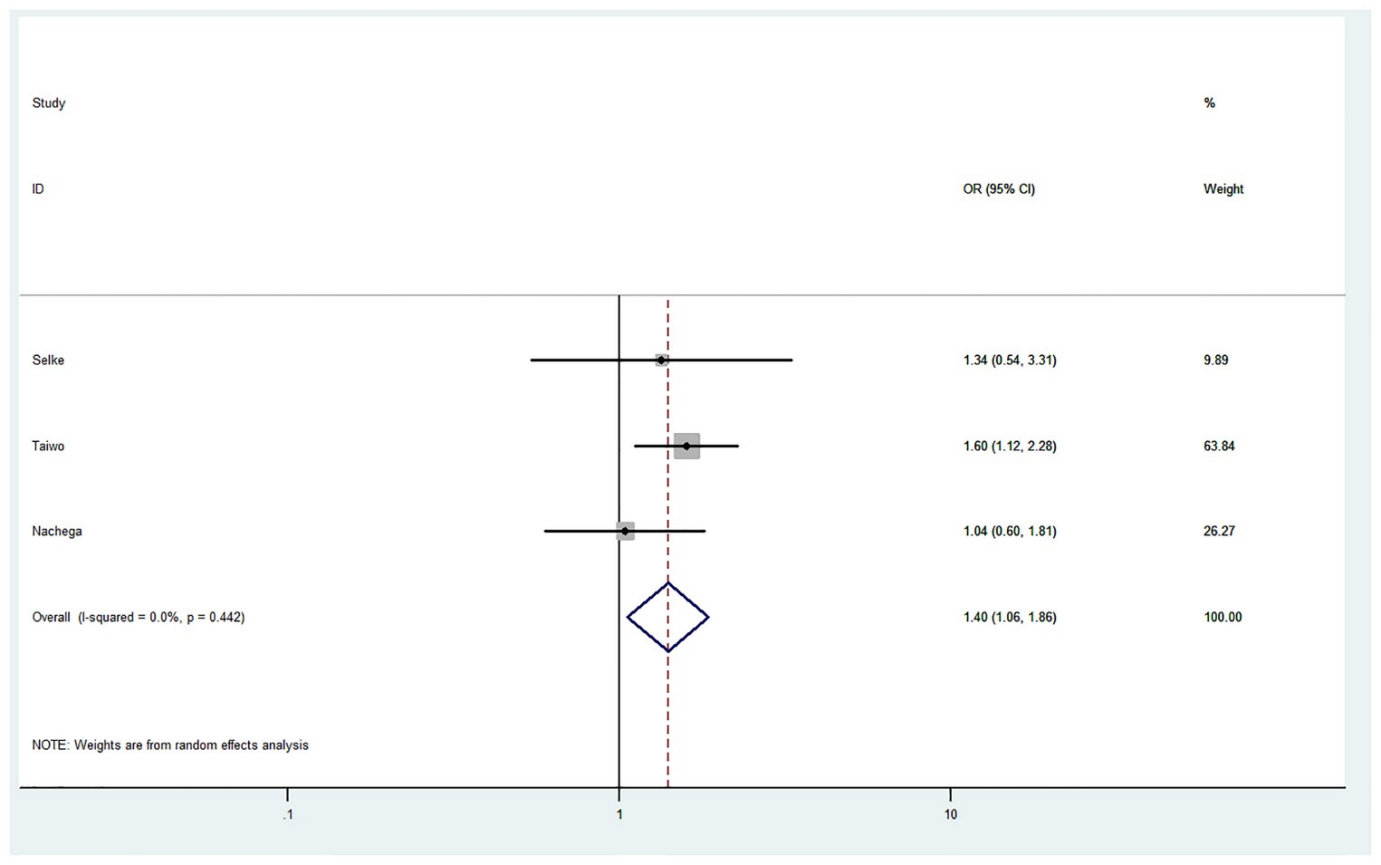
Pooled estimate for the impact of community health workers/lay workers on viral suppression. The circles represent the point estimate for viral suppression, the size of the grey squares around each point estimate reflect the weight of each study in the average effect size. The horizontal lines represent 95% confidence interval for the point estimate. The diamond represents the weighted average point estimate.

For outcomes that were consistently documented, we performed a pooled analysis. The pooled estimate for the impact of community health care workers/lay workers or peers on viral suppression monitored via viral loads was 1.40 [95% CI 1.06–1.86] (Fig 3).

### Quality assessment/risk of bias within and across studies

**Fig 4:**
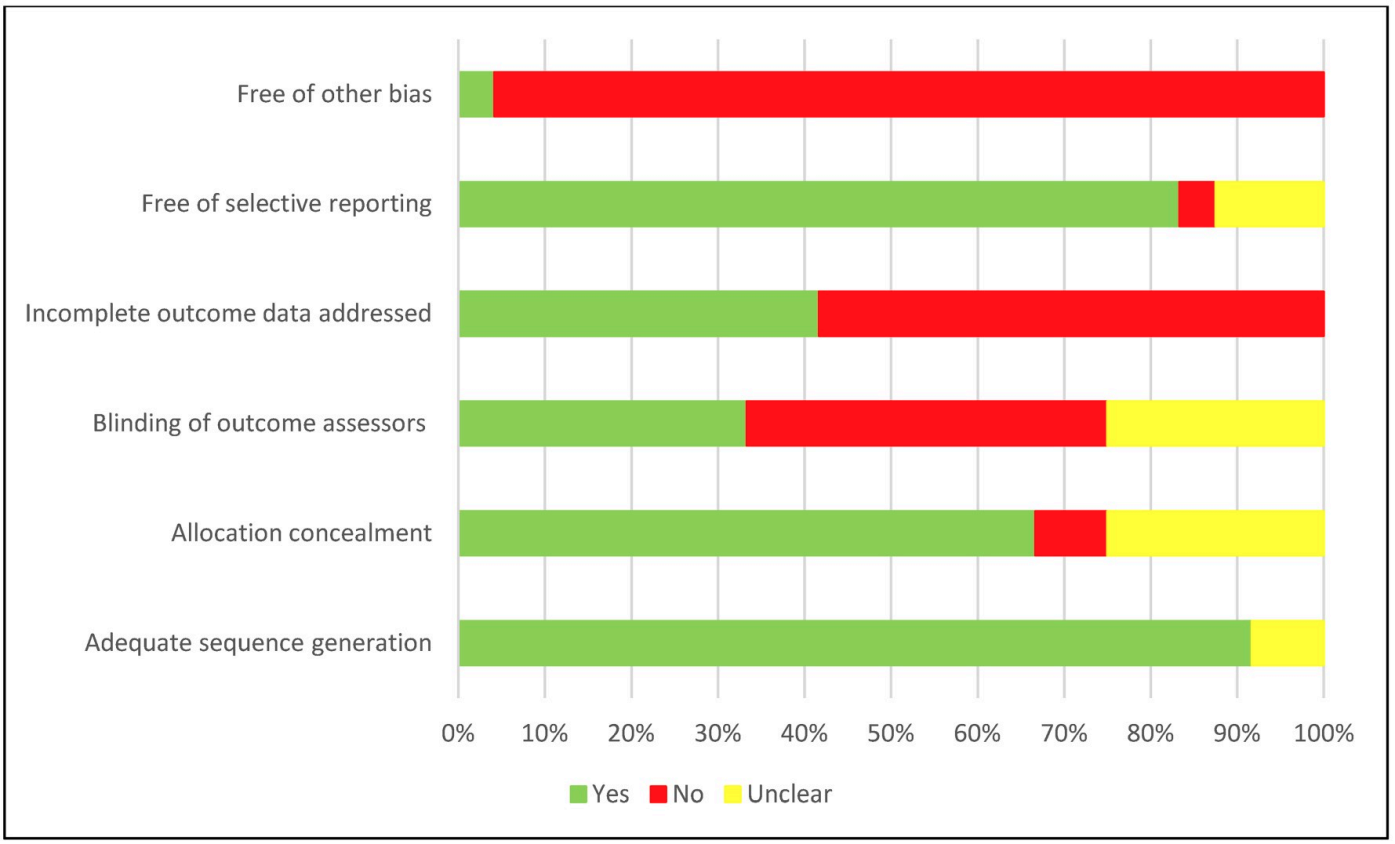
Risk of bias assessment for RCTs.

Among the randomised control trials (Fig 4), while a majority of the studies performed well on adequate sequence generation and allocation concealment, blinding of outcome assessors, treatment of missing/outcome data and other biases (such as recall bias, misclassification bias, social desirability bias, etc) were of concern.

**Fig 5:**
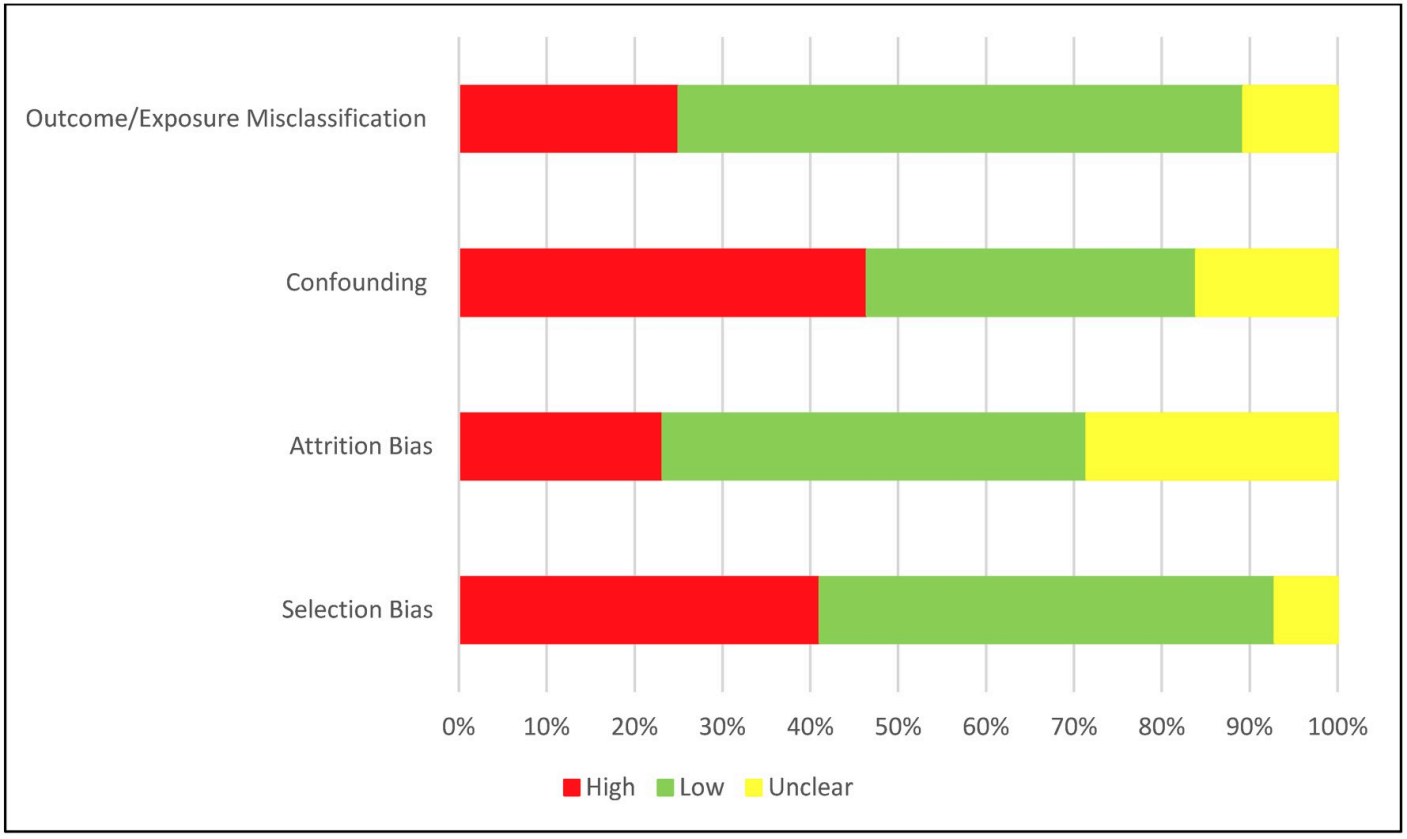
Risk of bias assessment for observational studies.

Overall, a vast majority of the studies were observational, in particular, confounding, selection of participants and information bias were of concern. Such biases need to be minimised when determining the true effect of any intervention (Fig 5).

## Discussion

### Summary of findings

Our systematic review aimed to collate community-based interventions that were successful in reaching the UNAIDS 90-90-90 targets. By categorizing interventions based on success, intervention type and stratifying by country income level, our aim was to provide information on interventions that are successful in contributing towards the UNAIDS 90-90-90 targets, such that researchers, policymakers can find value in and potentially implement modified interventions and policy changes that could work in comparable settings. The results of our review are aligned to previous reviews [14–16, 18, 19] but are novel in that they indicate a new emerging trend: in addition to the use of community health workers to achieve success across all targets, the use of technology/digital innovations and test/treat interventions can also supplement efforts to reach the second and third 90.

Overall, data on the first and third 90 were strong, while data on the second 90 requires work. A greater number of initiatives focused on the first target, compared to targets 2 and 3 (increasing linkages to care or attaining viral suppression). According to the UNAIDS report, a greater focus on linkages was needed, and this seen in our review [7]. In particular, among the successful interventions, the use of community health workers (peers, lay workers, directly observed therapy, etc) was commonly deployed (25/55, 45.5%) showing positive effects across the three targets, especially in lower middle and low-income countries.

Community-based interventions demonstrated success when their implementations were tailored to country or community context. For example, in a country or community where multiple concurrent partners were common, the couples voluntary HIV counselling and testing was not an ideal way to increase test uptake [73]. Integrated or combined test/treat strategies were the next most common interventions used with positive results (13/55, 23.6%). HIV campaigns were common for testing and treatment, mobile interventions were commonly used for HIV testing and educational programs and digital strategies (technology interventions) deployed for viral suppression. Data from low and middle-income countries, with high HIV burden, dominated the data.

For the first 90, across all income levels, decentralized testing initiatives like mobile testing and home-based HIV testing and counselling services were able to reach vulnerable populations that may not have presented themselves to healthcare facilities for testing. Testing initiatives by members of a community (such as community health workers, lay workers, link workers, etc) and those familiar to the patients resulted in a much higher uptake of testing; this was especially true in middle and lower income countries [21–23, 25–27, 74]. Similarly, mobile testing in a non-clinical setting also proved to yield far greater numbers of individuals being tested than in a clinical setting [35–37]. Decentralized and mobile testing strategies were successful in reaching younger, hard to reach populations [75–78].

For the second 90 (linkage to care/on treatment), community health workers/lay workers were most successful in facilitating linkages to care. Of nine studies, three did not show a positive effect with linkages to care; 2 studies reached targets for HIV testing, yet linkage to care/initiation of ART lagged, even with community HIV care providers [23, 26]. Combined test and treat interventions with creative integration services into antenatal care, for example, were successful in reaching the second target. Of note, none of the mobile testing interventions yielded success towards the second 90 [35, 37, 79], indicating that while mobile testing initiatives can largely increase testing uptake among hard-to-reach populations, linkages need to be designed, keeping the needs of the populations in mind. Communication of results is facilitated by these interventions, and that can improve their success rates.

Despite the WHO’s recommendation on the implementation of ART for all HIV-infected people regardless of CD4 count, many lower income countries face limitations (i.e, financial, infrastructural and consistent availability of ARTs), that impedes success towards the second 90 [80]. While the feasibility of providing CD4 counts onsite is heavily dependent on the economic context, having both CD4 count testing and ART initiation services available at the same time and location is effective in helping reach the second 90 target [35, 47]. In particular, same day HIV testing and ART initiation has shown to improve retention in care and subsequent virologic suppression [8].

Regarding the third 90, nuanced evidence in support of the integration of linkage to care services with micro-clinics and informal social networks which are normally used for the management and prevention of chronic disease, can increase linkages and subsequent viral suppression [81]. Task shifting to home visits has improved adherence but findings need to be further confirmed in larger, well designed studies. Task-shifting is appropriate in reducing the burden at hospitals or clinics and can reach larger populations [82, 83]. However, the spacing of home visits may have influenced patients’ adherence; more frequent visits may potentially close this gap. Task shifting in every aspect of the care continuum is not only cost effective but reduces barriers that vulnerable populations face especially in middle and lower income countries [55, 57, 84].

Digital technology has become a prominent intervention in increasing ART adherence; this includes daily text messages, beepers and alarm devices that remind patients to take medications. Three studies (37.5%) using digital technology did not show positive outcomes. Nonetheless, the use of technology to improve ART adherence and viral suppression is a good alternative to the use of community health workers where patients may feel their autonomy is violated.

Text message-based programs in low and middle-income populations with high rates of mobile phone usage may improve test uptake, treatment adherence rates, and increase viral suppression, but frequency of text messages heavily influences adherence levels [85]. Furthermore, these messages have the potential to consider the psychosocial aspect of ART intake and potentially builds a relationship between patient and provider [71] if text messages are tailored. Similarly, educational interventions have also been successful in improving treatment adherence, but are more so a valuable component when integrated with other interventions.

In summary, using the UNAIDS 90-90-90 targets as a framework creates momentum in our populations to reach the goals, furthermore, if studies reported according to the targets, using the metrics most commonly used, direct comparisons of intervention effectiveness can be made.

### Strengths

This systematic review provides condensed information regarding interventions successful towards each of the 90-90-90 targets within income levels and by UNAIDS targets. Strategies must be multifaceted and designed to recognize the social context of the population in question. The decentralization of testing to familiar, comfortable and non-clinical settings using peer support and community workers has been demonstrated to improve testing uptake and linkage to care rates in key populations, such as men who have sex with men, female sex workers, and injection drug users.

Direct comparisons of interventions and their ability to reach the UNAIDS targets is difficult given inconsistent reporting. Metrics used to measure HIV testing, linkage to care and viral suppression must be streamlined and effectively used across interventions looking at these outcomes such that we can make direct comparisons of the effectiveness of interventions and inform the scale up of them.

We have shown that reporting outcomes post 2014 will need to continue to follow the UNAIDS targets as these targets are aligned to the HIV care continuum, which will ultimately help us compare interventions and determine success.

### Limitations

This review is unique in its framing of outcomes aligned to UNAIDS 90-90-90 targets globally [14–16, 18, 19]. Our study stands out in that we have not limited the outcomes nor excluded populations.

As with all systematic reviews, our study has limitations. Although an exhaustive literature search was carried out, by also including hand searched articles, our search strategy may have missed some articles and interventions. Furthermore, majority of the studies were observational and at high risk of various bias such as confounding and selection bias, thus decreasing our confidence in reported intervention effects. Additionally, due to the limited number of studies included in the meta-analysis, the potential risk of publication bias could not be assessed. A fewer number of studies results in a lower power of the tests to distinguish chance from real symmetry [86]. Despite this, publication bias is a likely risk in this situation. Future systematic reviews and meta-analyses regarding community-based interventions and its effect on the UNAIDS 90-90-90 targets should consider assessing publication bias. Another limitation is that studies included in this review have shown variation in metrics used to measure all three targets. Some studies measured the first 90 by reporting only the number of people that tested or reporting first time testers, or an increase in HIV testing, but did not report a denominator, thus proving difficult to determine if an intervention is effective. Metrics to measure linkage to care also varied across studies; it was unclear whether metrics involved ART initiation or simply healthcare provider appointments to seek further care among others. Studies reporting on the third 90 used metrics such as viral loads or CD4 counts. Approximately 50.0% of the studies reporting on testing, linkage to care or viral suppression reported using metrics aligned to the targets. Additionally, some studies lacked a comparator group and among others, there was little consistency across populations, outcome metrics and interventions limiting the assessment of pooled effectiveness of interventions to its fullest extent. Furthermore, future reviews can focus on investigating, in more detail, successful interventions within specific regions that may be effective at any point within the cascade of care. This may allow for more pooling of studies to determine intervention effects.

Despite these limitations, this review draws strength from including over 80 studies, representing different interventions, populations and regions, that focused on various aspects of the UNAIDS targets, and also focused on other interventions that can help meet those targets directly or indirectly.

## Conclusion

Use of community health care/peer workers is strongly recommended. Exploration of innovative interventions for linkage to care and to engage first time testers is encouraged. Besides, reporting of studies aligned with UNAIDS targets is recommended. Stratification by targets and country income levels is informative, and guides adaptation of successful interventions in comparable socio-economic settings. Consistent reporting of clear metrics aligned to UNAIDS targets is vital to informing policy initiatives. Finally, with 2020 fast approaching, resources need to be targeted to achieve the ambitious UNAIDS targets. Including reporting of studies by UNAIDS targets simplifies policy comparisons and determination of success towards the targets. This in turn could potentially catalyze greater progress towards the UNAIDS 90-90-90 targets.

## Supporting information

S1 Checklist PRISMA Checklist. (DOC)

S1 Table Summary of study characteristics. (DOCX)
