# Supplementary material for: Which community-based HIV initiatives are effective in achieving UNAIDS 90-90-90 targets? A systematic review and meta-analysis of evidence (2007-2018)
Source: PLoS One. 2019 Jul 17;14(7):e0219826. doi: 10.1371/journal.pone.0219826 (PMC6636761; doi:10.1371/journal.pone.0219826)
Supplement: S1 Table — (DOCX) [file pone.0219826.s002.docx]

Community Health Workers/Peers, etc.

| No. | Author | Study Design | Participants/Country | Intervention | Measure/Metric | Results (MD, mean difference; RR, risk ratio; IRR, incidence rate ratio; HR, hazard ratio; SD, standard deviation, 95% CI when presented; Y, years; M, months; W, weeks) | 90-90-90 Target |
| --- | --- | --- | --- | --- | --- | --- | --- |
| 1 | Fair, 2014 | Longitudinal study | High-risk population of youth, HIV+, **USA**  n = 15 | Use of social worker in HIV/AIDS pediatric program to increase adherence and care. Interventions included daily phone calls/ texts, home visits, reward system for increased CD4 or decreased VL based on lab tests were given to youth. | ART adherence:  CD4 count | CD4 counts were significantly higher [t(14) = −2.35, p = 0.03] / Effective | 3^rd^ |
|  |  |  |  |  | ART adherence: VL | VL were significantly lower at the most recent clinic visit [t(14) = 2.08, p = 0.05] / Effective | 3^rd^ |
| 2 | Becker, 2014 | Feasibility study | 15-49yrs, Heterosexual couples, **Malawi**  n = 48 (CHCT + CFP)  n = 97 (CHCT)  n = 4 (CFP) | Male and female counselors visited each couple to increase HIV testing and counseling. Pre-test counselling given to couples who consented to both CHTC and CFP, rapid HIV test administered, CFP services and condoms offered in the 20–40 minutes while awaiting test results. | Testing: Attendance rate | 89% of couples accepted at least one of the services. First-ever HIV testing was delivered to 25 women and 69 men, resulting, respectively, in 4 and 11 newly detected infections. 65% of those in CHCT+CFP and 61% of participants spoke to partners about HIV testing / Effective | 1^st^ |
| 3 | Markwick, 2014 | Feasibility study | HIV-, IDU, **Canada**  n = 600 | Peer-delivered voluntary counselling and testing (VCT) in Vancouver, Canada. Survey questions asked respondents who they would want to administer HIV pre-test counselling, rapid HIV test, and HIV post-test results and counselling. | Acceptability: Self-reported | 309 individuals (51.5%) indicated willingness to receive peer-delivered pretest counselling, 244 (40.7%) to receive peer-delivered rapid HIV testing, and 257 (42.8%) indicated willingness to receive peer-delivered post-test counselling/ Moderately feasible. | 1^st^ |
| 4 | Hickey, 2015 | Quasi-experimental | HIV+, on ART, **Kenya**  n=153 (Int)  n=216 (Ctrl) | Microclinic Social Network intervention on engagement in HIV care and medication adherence | Testing: follow-up appointment | 11% of intervention arm participants arm and 20% of those in the control arm experienced a clinic absence of ≥ 90-days | 1^st^ |
|  |  |  |  |  | ART adherence: Concentration of ART (NVP) in hair | 111/153 and 162/216 were on NVP in intervention and control group respectively. MD of NPV in hair decreased in both groups from baseline to 6M: −5.5, SD 42.4 in intervention and −12.4, SD 38.8 in control. | 3^rd^ |
| 5 | Plax, 2015 | Cross-sectional | ≥13yrs, Missouri, **USA** | Supporting Positive Opportunities with Teens (SPOT), a community-based health and social service facility for increasing STI testing and linkage to care among youth and hard to reach populations. | Testing: Attendance rate | 5703 HIV tests performed between 2008-2013. 59 (1%) were HIV+ | 1^st^ |
|  |  |  |  |  | Linked to care | Newly diagnosed HIV+ youth referred to Linkage to Care Case Manager, 80% met with case managers for further support, care and services. | 2^nd^ |
| 6 | Root, 2017 | Feasibility | HIV+, client of Shiselweni Home-Based Care Organization (SHBC), **Swaziland**  n = 79 | Home based HIV prevention and treatment support services offered by SHBC with faith-based component | Feasibility: self-reported | 92% believed health improved since care supporter began home visits. | Other |
|  |  |  |  |  | Testing: Attendance rate | 27.9% tested for HIV after home visits. 31% started ART / Less feasible. | 1^st^ |
| 7 | Sheneberger, 2017 | Feasibility | **Zambia** | Community HIV Epidemic Control (CHEC) Model of University of Maryland. Community health worker with tablet linked to national electronic health record offers HIV testing and promotional health messages to members of the community. Those tested +ve are linked to care. | Feasibility: testing | HIV testing rose from 21051 to 71289 (339% increase). | 1^st^ |
| 8 | Naik, 2015 | Prospective cohort | 15yrs+, HIV+, **South Africa**  n = 492 | Part of larger RCT “Good Start” d lay counsellors offered free rapid HIV testing to all consenting household members aged 18 years or older and to those aged 14 to 17 years with parental or guardian consent. | Linked to care | Within 542 days after home-based HIV counseling and testing (HBHCT), 76.0% (95% CI: 71.6 to 80.4%) had a final outcome of linkage to care, 62.1% within three months (95% CI: 55.7 to 68.5%). | 2^nd^ |
|  |  |  |  |  | ART adherence: CD4 | Of those linked to care, 82.8% had a recorded CD4 count with median 341 cells/mm3 (IQR 224 to 542 cells/mm3) | 3^rd^ |
| 9 | Hayes, 2017 | Cross-sectional | 18–44yrs, **Zambia**  n=121,130 | PopART intervention comprises annual rounds of home-based HIV testing delivered by community HIV-care providers | Testing: home based testing | 80% of men and 85% of women knew their HIV status after the community HIV-care providers visits. | 1^st^ |
|  |  |  |  |  | Linked to care: ART initiation | 6,197 HIV-positive adults referred by CHiPs, 42% (95% CI: 40%–43%) initiated ART within 6M and 53% (95% CI: 52%–55%) within 12M | 2^nd^ |
| 10 | Carrico, 2014 | Cross-sectional | Methamphetamine-using MSM  n = 123 | Stonewall Project Model delivers harm reduction and substance management to those in need. It also delivers sexual risk reduction interventions. | Linked to care: On ART | For baseline, 6M and 12M follow-up, number and % of those on ART was 33 (46.5%), 40 (58%) and 29 (47.5%). Cohen's h = 0.02 / Effective. | 2^nd^ |
|  |  |  |  |  | ART adherence: VL | For baseline, 6M and 12M follow-up, number and % of those with undetectable VL was 14 (20.0%), 24 (42.1%) and 22 (37.3%). Cohen's h = 0.38, p<0.01. / Effective | 3^rd^ |
| 11 | Washington, 2014 | Cross-sectional | FSW, **India**  n = 14 284 | Comprehensive HIV prevention intervention directed towards FSW for outreach, medical and referral services via the use of link workers. | Testing | 6428/14282 ever had HIV testing in the preceding 6M. Significant increase in HIV testing of FSW from 2009 to 2011 (p<0.001), 29.6% to 94.7% respectively. / Effective. | 1^st^ |
| 12 | Beeson, 2016 | Cross-sectional | **South Africa**  n = 2898 | 11 community health workers performed screening in traditional Zulu homes; TB symptom screening, non-communicable disease screening and HCT services. | Testing: first time testers | 1470 agreed to HCT. 27.4% of those agreeing to HCT reported never testing 46 were newly diagnosed. | 1^st^ |
|  |  |  |  |  | Linked to care | 32/46 were linked to care with medium linkage time of 3W. 23/32 initiated ART. | 2^nd^ |
| 13 | Tibenderana, 2016 | Cross-sectional | **Uganda**  n = 14 villages | STAR-EC is an 8Y district based project, where community quality improvement teams promote facility community linkages and retention on ART.14 low performing villages were selected to partake in the study. | Linked to care: Follow up appointment | Clinic appointment keeping increased from 85% to 96% over 2Y period. | 2^nd^ |
|  |  |  |  |  | Linked to care; self reported | Linkage to care increased from 50% to 87% over 2Y | 2^nd^ |
|  |  |  |  |  | ART adherence: self-reported | Retention on ART increased from 70% to 90% over 2Y. | 3^rd^ |
| 14 | Casavant, 2016 | Feasibility | 15-59yrs, residents of Chokwe district, **Mozambique**  n = 53277 | Home based HIV counselling and testing offered to all residents. | Testing | 25344 / 53277 (47.6%) tested for HIV. Of those tested, 20.2 were HIV infected and 7.75 were newly diagnosed. | 1^st^ |
| 15 | Manjezi, 2016 | Cross-sectional | **South Africa**  n = 14779 index clients, 66766 household members | Home based HIV testing and counselling done by lay community health workers at residents of index clients (HIV+ clients) | Testing | 59457 / 66766 (89%) received HIV testing. 9219 were HIV+. 70 / 2837 children were HIV+ | 1^st^ |
|  |  |  |  |  | Linked to care | 8642/9219 (93.7%) were successfully linked to care. 100% of children were linked to care. | 2^nd^ |
| 16 | Asiimwe, 2016 | Cross-sectional | **Uganda**  n = 23940 | The use of Community Health Extension Workers (CHEWs) to accelerate community access to integrated testing for HIV, malaria and Hep B. | Testing | 23940 were tested through CHEWs whereas only 3884 were tested at district health facilities for the period Oct – Dec 2015, covering the same areas. 498 / 23940 were HIV+ and 217 / 3884 were HIV+ tested from health facilities in Sheema. | 1^st^ |
|  |  |  |  |  | Linked to care | 63.1% were linked to care via CHEWs whereas 14.2% were linked to care who tested at health facilities. | 2^nd^ |
| 17 | Selke, 2010 | RCT | HIV+, ≥18 yrs, stable on ART for at least 3M, **Kenya**  n = 87 (Int)  n= 102 (Cntrl) | Using PLHIV as community care coordinators to do monthly home visits to assess patient symptoms, vital signs, adherence to ART and opportunistic infection prophylaxis. | ART adherence: CD4 and VL | At 6M mean CD4 cell count was 354 (232-451) and 306 (214-410) for intervention and control respectively, p=0.24 (95%CI -19, 74). At 12M mean CD4 cell count was 404 (265-527) and 358 (240-522) for intervention and control respectively, p=0.50 (95%CI -38, 77). / No impact  Detectable VL at 12M , 9 (10.5%) vs. 13 (13.5%) in intervention vs. control groups, p=0.42 95%CI 0.37, 1.34 / No impact | 3^rd^ |
| 18 | Lugada, 2010 | RCT (sub study of Cluster randomized trial) | Household members of HIV patients on ART, **Uganda**  n=859 (Int – home VCT), n=594 (Cntrl – Clinic VCT) | Comparison of home based and clinic based VCT. In home based VCT, lay filed officers visited houses of HIV patients on ART and offered services to all members. | Testing | Persons in the home arm were much more likely to undergo HIV testing and receive their results than persons in the clinic arm [adjusted odds ratio (aOR) 10.41; 95% CI: 7.89 to 13.73; P , 0.001], trial went on for 2Y. / Effective | 1^st^ |
| 19 | Kipp, 2010 | Non-randomized cohort study | >18yrs, People living with HIV, **Western Uganda**  n = 185 community based;  n = 200 hospital cohort | Weekly home visits to patients by trained volunteers who delivered antiretroviral drugs (ARVs), monitored and supported adherence to treatment, and identified and reported adverse reactions and other clinical symptoms. Control was a hospital-based cohort who received standard care | Viral suppression; VL | Successful ART treatment outcome as measured by virological suppression (VL<400 copies/ml) in the CBART cohort was similar to those in the hospital-based cohort (90.1% vs 89.3%, p=0.47). The median CD4 cell count increased significantly in both cohorts (community-based cohort 159 cells/μl vs 145 cells/μl in the hospital-based cohort)./ No impact | 3^rd^ |
| 20 | Fatti, 2012 | Observational multicohort study | >16 yrs, HIV+, **South Africa**  n = 19,668 (CBAS) n = 47,285 (control) | Community-based adherence-support (CBAS) useS patient advocates (PAs). PAs who provide adherence and psychosocial support for ART patients, and undertake home visits to ascertain and address household challenges potentially impacting on adher- ence. PA support starts from the time of pre-ART preparation and continues throughout long-term patient care. | Viral suppression: VL | After 6M of ART, virological suppression was 76.6% (95% CI: 75.8% to 77.5%) in CBAS patients versus 72% (95% CI: 71.3% to 72.5%) in non-CBAS patients (P , 0.0001), adjusted odds ratio was 1.22 (95% CI: 1.14 to 1.30). Improvement in virological suppression occurred progressively for longer durations of ART [adjusted odds ratio was 2.66 (95% CI: 1.61 to 4.40) by 5 years]./ Effective | 3^rd^ |
| 21 | Taiwo, 2010 | RCT | > 18 yrs, HIV +, **Nigeria**  n = 248 (Int TPA+SOC) n = 251 (Cntrl - SOC) | Intervention group received SOC and chose a partner who attended one adherence education session. Partners were to aid in overall patient adherence. | Viral suppression: VL | Undetectable VL was achieved by 61.7% of patients in the TPA (Treatment partner assisted) arm versus 50.2% of those receiving SOC (standard of care) [odds ratio (OR) = 1.58, 95% CI: 1.11 to 2.26, P , 0.05]./ Effective | 3^rd^ |
|  |  |  |  |  | ART adherence: CD4 | No significant differences in CD4 cell count increases (t = 20.09, df = 404, P . 0.05). / No impact | 3^rd^ |
|  |  |  |  |  | ART adherence: pill count | The TPA group had more than 3 times the odds of at least 95% drug pickup adherence through week 24 (OR = 3.06, 95% CI: 1.89 to 4.94, P , 0.01) and almost twice the odds through week 48 (OR = 1.95, 95% CI: 1.29 to 2.93, P , 0.01). / Effective | 3^rd^ |
| 22 | Chang, 2010 | RCT (Cluster-randomized trial) | AIDS clinics, **Uganda**  n = 10 (peer health workers) n = 5 (control) | PHW recruitment, training, tasks, and monitoring was developed, but the intervention was allowed to adapt to needs and problems which arose, e.g. arranging for a home visit to occur at a worksite if so requested by a patient. | ART adherence: pill count | No significant differences were found in lack of adherence (,95% pill count adherence risk ratio [RR] 0.55, 95% confidence interval [CI] 0.23–1.35; ,100% adherence RR 1.10, 95% CI 0.94–1.30) / No impact | 3^rd^ |
| 23 | Nachega, 2010 | RCT | >18 yrs, HIV+, **South Africa**  n = 137 (intervention) n = 137 (control) | Directly observed therapy (DOT) for antiretroviral therapy (ART). Treatment-supporter DOT-ART or self-administered ART. DOT- ART patients and supporters received baseline and follow-up training and monitoring. | ART adherence: VL | Vl < 400 copies/ml at 12M were 72.8% in the DOT-ART arm and 68.4% in the Self-ART arm (P 1⁄4 0.42). DOT-ART patients had greater median CD4 cell count (cells/ml) increases at 6 months [148 (IQR 84–222) vs. 111 (IQR 44–196) P1⁄40.02] but similar results at all other time-points. Survival was significantly better in the DOT-ART arm (9 deaths, 6.6%) than in the Self- ART arm (20 deaths, 14.6%; log-rank P 1⁄4 0.02) | 3^rd^ |
| 24 | Kang’ethe, 2009 | Feasibility | Community caregivers, **Botswana**  n=82 | Community primary caregivers were interviewed in focus groups to determine the acceptability of using traditional healers in a community home-based care program (CHBC). | Acceptability: self-reported | Traditional healers are important players in caregiving of persons with HIV. Caregivers indicated that inadequate faith in the biomedical referral system strengthens belief in traditional healers. Cultural acceptance of traditional healers suggests that their services are especially recommended by elderly. | 2^nd^ |
| 25 | Munoz, 2010 | Cohort study | HIV+, living in poverty, on HAART, **Peru**  n=60 (Int) n=60 (Ctrl) | Intervention group received community-based accompaniment with supervised antiretroviral (CASA) therapy by DOT workers, who were responsible for supervising all outpatient HAART doses in patients’ homes or elsewhere if by the patient over 12M. | Viral suppression: VL | CASA participants were more likely to remain on HAART at 12 months (90.0% vs. 65.0%, Fisher’s (2, N = 120) = -0.0001, P<0.01) , and adhere to HAART (80.0% vs. 61.7%, V2 (1,N = 120) = 4.88, P<0.05). Participants were more likely to have a suppressed viral load at 12 months if they received CASA support (76.7% vs. 58.3%, V2 (1, N = 120) = 4.60, P<0.05). | 3^rd^ |
| 26 | Nyamathi, 2012 | RCT | HIV+, women, 18-45 yrs, ART for 3M, **India**  n=34 (Int) n=34 (Ctrl) | Accredited Social Health Activists (ASHAs) to improve antiretroviral therapy adherence with visits 4-5 times/week to monitor ART adherence of rural women living with AIDS in India compared to that of a usual care group. | ART adherence: pill count & CD4 | After 6M, while both groups improved their adherence to antiretroviral therapy, there was greater improvement in the Asha-Life group (p < 0.001), who reported a greater reduction in barriers to antiretroviral therapy than those in the usual care group./ Effective | 3^rd^ |
| 27 | Pearson, 2007 | RCT | HIV+, ≥18 yrs, **Mozambique**  n=175 (Int) n=175 (Ctrl) | Participants assigned to receive 6W (Monday through Friday; 30 daily visits) of peer-delivered, modified directly observed therapy (mDOT) or standard care. Peers provided education about treatment and adherence and sought to identify and mitigate adherence barriers. | ART adherence: self-reported & CD4 | Intervention participants, compared to those in standard care, showed significantly higher mean medication adherence at 6M (92.7% vs. 84.9%, difference 7.8, 95% confidence interval [CI]: 0.0.02, 13.0) and 12M (94.4% vs. 87.7%, difference 6.8, 95% CI: 0.9, 12.9). / Effective  There were no between-arm differences in chart-abstracted CD4 counts. | 3^rd^ |
| 28 | Sarna, 2008 | RCT | HIV+, >18 yrs, ART naïve, **Kenya**  n=116 (Int) n=118 (Ctrl) | Patients received modified directly observed therapy (mDOT) services for the first 24 weeks of ART or standard care. Follow-up continued until week 72. | ART adherence: pill count | During weeks 1-24, 9.1% (9/99) of m-DOT participants reported missing doses compared with 19.1% (20/105) of controls (P = 0.04) and 96.5% (517/571) of m-DOT pill-count measures were ≥95% compared with 86.1% (445/517) in controls [adjusted odds ratio = 4.4; 95% confidence interval (CI) = 2.6 to 7.5; P < 0.001. Adherence with m-DOT was 4.8 times greater (95% CI = 2.7 to 8.6; P < 0.001) with adjustment for depression and HIV-related hospitalization./ Effective | 3^rd^ |
| 29 | Mugusi, 2009 | RCT | HIV+, ≥18 yrs, eligible for ART, **Tanzania** n=312 (regular counseling only) n=242 (regular counseling and calendars) n=67 (treatment assistants) | Patients were randomly assigned to either regular adherence counseling, regular counseling plus a calendar, or regular counseling and a treatment assistant and seen monthly. | ART adherence: self-reported & CD4 | In 94.8% of all visits, patients reported to have adhered to treatment. In only 39 (0.7%) visits did patients report a <95% adherence. There were no differences in adherence (P = 0.573) or differences in CD4 count and weight changes over time in the interventions. / No impact | 3^rd^ |
| 30 | Coates, 2014 | RCT | Communities; **South Africa, Tanzania, Zimbabwe, Thailand**  n=25 (Int) n=25 (Cntrl) | Communities were randomized to receive community-based voluntary counselling and testing (CBVCT) or standard voluntary counselling and testing (SVCT). Data were collected and baseline and post-intervention by surveys | Testing | Overall 12M testing rates (Table 7) were 25% higher in the CBVCT arm than in the SVCT arm (95% CI 12%-39%, p=0·0003). Testing rates over 36 months were 27% higher in the CBVCT arm (95% CI 15%-41%, p<0·0001). / Effective | 1^st^ |

Educational

| No. | Author | Study Design | Participants/Country | Intervention | Measure/Metric | Results (MD, mean difference; RR, risk ratio; IRR, incidence rate ratio; HR, hazard ratio; SD, standard deviation, 95% CI when presented; Y, years; M, months; W, weeks) | 90-90-90 Target |
| --- | --- | --- | --- | --- | --- | --- | --- |
| 31 | Rios-Ellis, 2015 | Quasi-  experimental | ≥18yrs, Latino/Hispanic, **USA**  n = 579 | 60-90min interactive group education held by *promotores*(who are HIV+, or affected) at various locations emphasizing on Latino culture, HIV/AIDS transmission, prevention, testing, etc | ATT testing: Self-reported | On scale of 1-5 (1 low, 5 high) MD in willingness to seek HIV testing in next 3M was 0 (4.3 95% CI 4.2-4.2 and 95% CI 4.3-4.4 for pre- and post-test, respectively) / No impact | 1^st^ |
| 32 | Schulden, 2013 | Uncontrolled trial | ≥13yrs, HIV-, **USA**  n = 5247 | 3 community-based organizations offered rapid HIV testing via various outreach to increase HIV testing targeting migrant communities. | Testing: first-time testers | 59% of people who obtained rapid HIV test were first time testers. (65.5% males and 48.4% females). 0.1% were HIV+ | 1^st^ |
|  |  |  |  |  | Feasibility: Completion rate | 3135/5247 who tested completed survey on socio-demographic characteristics, HIV/STD risk factors, HIV testing and immigration history./ Highly feasible | 1^st^ |
| 33 | Khachani, 2012 | Cohort study | HIV+, ART at least 2M, **Morocco**  n=50 | Patients received 3-5 one-hour personalized educational and psychological support sessions. | ART adherence: VL CD4 | The medians of adherence scores showed no change (P=0.266); they were already at the optimal level of 1 at baseline and remained so throughout the study. Patients with an undetectable viral load were 52% at baseline / No impact | 3^rd^ |
| 34 | Siedner, 2012 | Cohort study | HIV+, initiating ART, **Uganda**  n=300 | Participants in the intervention group underwent pre-therapy adherence counseling, while the control group experienced counseling on or after initiation of ART. | Linked to care; Electronic records | Participants who completed pre-therapy counseling visits had longer delays from ARV eligibility to initiation (median 49 vs. 14 days, p<0.01). In multivariable analyses, completing adherence counseling prior to ARV initiation was not associated with average adherence >90% (AOR 0.8, 95%CI 0.4–1.5), absence of treatment gaps (AOR 0.7, 95%CI 0.2–1.9), or HIV viremia (AOR 1.1, 95%CI 0.4–3.1)./No impact | 2^nd^ |
| 35 | Holstad, 2012 | Quasi-experimental | HIV+, women, on ART, **Nigeria**  n=30 (Int) n=30 (Ctrl) | Participants underwent a motivational intervention (MI) of 8 group sessions to enhance self-efficacy, HIV knowledge, ART adherence, and safer sex behaviors. Sessions lasted 1.5-2 hours. Control group sessions focused on general health education. | ART adherence: Self-reported | 100% of the MI respondents reported taking 100% of their HIV drugs compared to only 11 (61.1%) of the 18 control respondents who took 90% or more of their HIV drugs. On the single item adherence question, 93% of MI participants reported “never” missing any medications compared to 40% of the HPP group (χ2=15.777, df =1, p < .0001). / Effective | 3^rd^ |
| 36 | Safren, 2009 | RCT | HIV+, depressed, **USA**  n=21 (Int) n=22 (Ctrl) | Cognitive–behavioral therapy to enhance medication adherence and reduce depression (CBT-AD). | ART adherence: MEMs | Those who received CBT-AD evidenced significantly greater improvements in medication adherence and depression relative to the comparison group (F(1, 42)=21.94, p<0.0001). Those who were originally assigned to the comparison group who chose to cross over to CBT-AD showed similar improvements in both depression and adherence outcomes. / Effective | 3^rd^ |
| 37 | Becker, 2010 | RCT | HIV+, women, **Tanzania**  n=761 (IVCT) n=760 (CVCT) | Participants were randomized to either voluntary counseling and testing (VCT) for couples (CVCT) or individuals (IVCT) to compare their acceptance and effectiveness. | Testing | Among the 761 women randomized to receive IVCT, 704 (93%) agreed to counseling, 595 (78%) to testing, and 538 (71%) to receive results. Among the 760 women randomized to CVCT, only 254 (33%) returned with their partners, 115 (15%) returned alone and 391 (51%) did not return at all. | 1^st^ |
| 38 | Loggerenberg,  2015 | RCT | HIV+, ART eligible, **South Africa**  n=150 (didactic counseling prior to ART) n=147 (intensive motivational adherence intervention after initiating ART) | Participants either underwent didactic counselling of 3 sessions or intensive individualized motivational counselling using a client-centred approach. Counselling was tailored to specific participants needs to promote supportive, individualized care. | Viral suppression: VL | Virologic suppression at 9M was achieved in 89.8% of didactic and 87.9% of motivational counselling participants (risk ratio [RR] 0.98, 95% confidence interval [CI] 0.90-1.07, p=0.62) / No impact | 3^rd^ |
|  |  |  |  |  | ART adherence: pill count | 82.9% of didactic and 79.5% of motivational counselling participants achieved >95% adherence by pill count at six months (RR 0.96, 95%CI 0.85-1.09, p=0.51). Participants receiving intensive motivational counselling did not achieve higher treatment adherence or virological suppression than those receiving routinely provided didactic adherence counselling. / No impact | 3^rd^ |
| 39 | Sampaio-Sa, 2008 | RCT | HIV+, ART naïve, **Brazil**  n=52 (Int) n=55 (Ctrl) | Participants underwent an information-motivation-behavioral (IMB) skills program with counseling sessions lasting 2-3 hours each, administered by a psychologist and a social worker. The control group participated in four 8–12-min video-education sessions. | ART adherence: | After 3–6M, ART adherence was 77.8% in the workshop group and 85.7% in video group (as treated) and 53.8% and 65.5%, respectively, using intention-to-treat (ITT) analysis (both P[0.05) At 9–12 months, ART adherence decreased to 73.7% in the workshop group and 79.1% in the video group (as treated) and 53.8% and 61.8% using ITT, respectively. / No impact | 3^rd^ |
| 40 | Nkengfack, 2014 | RCT | HIV+, 20-72 yrs, ART naïve, **Cameroon**  n=100 (Int) n=101 (Ctrl) | An HIV-Care-Program, focusing on nutrition and lifestyle, which can be provided at scale to HIV-infected patients, on clinical and anthropometric parameters, and health status. | ART adherence: CD4 | After 6M, CD4 count dropped by 46.3 cells (7.7 %) (intervention) and 129 (23 %) (control) (p = 0.003). Mean time to ARV; 5.9 months 95 % CI (5.9, 6.0) (intervention); 4.9 months 95 % CI (4.7, 5.2) (control) (p<0.004). / Effective | 3^rd^ |

Integrated/combined testing and/or treatment services

| No. | Author | Study Design | Participants/Country | Intervention | Measure/Metric | Results (MD, mean difference; RR, risk ratio; IRR, incidence rate ratio; HR, hazard ratio; SD, standard deviation, 95% CI when presented; Y, years; M, months; W, weeks) | 90-90-90 Target |
| --- | --- | --- | --- | --- | --- | --- | --- |
| 41 | Anaya, 2015 | Quasi-experimental | Homeless Individuals, Los Angeles County, **USA** n = 817 | Testing was available to any adult residing in shelters during scheduled visits. Flyers and loudspeaker notifications were distributed and announced prior to visits. | Testing | During the 26 months of the project, counselors made 189 visits and administered 817 tests, 7/817 (0.86%) were HIV+. | 1^st^ |
|  |  |  |  |  | Linked to care: follow-up | 5/7 HIV+ individuals linked to care, 1 did not return for results, and 1 refused linkage. / Effective | 2^nd^ |
| 42 | Firestone, 2014 | Quasi-experimental | MSM, **Central America**  n = 520 | The Pan-American Social Marketing Organization (PASMO) and partners implemented combination package of prevention interventions and services: 1) behavioural, including interpersonal communications, and online outreach; 2) biomedical services including HIV testing and counselling and screening for STIs; and 3) complementary support. | Testing: Self-reported | 52% reported having had an HIV test in the last 12 months (Those exposed to behavioural interventions were more likely to have tested for HIV in the past year (AOR 1.76, 95% CI 1.01, 3.10). Exposure to the complementary component was also associated with HIV testing (OR 1.97; 95% CI 1.0, 4.05). AOR 2.98 (95% CI 1.82, 4.87) for exposure to any component of the combination package. / Effective | 1^st^ |
| 43 | Amnesty, 2015 | Feasibility study | ≥18yrs, New York **USA**  n = 332 | Examined in-pharmacy HIV testing among customers in pharmacies participating in a nonprescription syringe program in New York City. | Testing | 39.5% received in-pharmacy HIV testing. Being female (AOR=2.24; 95%CI 1.24–4.05), having multiple sex partners (AOR=1.20; 95% CI 1.06–1.35), having an HIV test more than 12 months ago (AOS=4.06; CI 1.85–8.91), injecting drugs in last 3 months (AOR=2.73; 95% CI 1.31–5.69) and having continuous care (AOR=0.32; 95% CI 0.17–0.58) were associated with receiving in-pharmacy HIV test. | 1^st^ |
| 44 | An, 2015 | Feasibility study | pregnant women, **Morogoro, Tanzania**  n = 196 | Implications for integrating HIV testing and counselling into maternal healthcare. | Acceptability: Self-reported | Perceptions of integrating HIV testing with routine antenatal care from women and health providers were generally positive. Respondents felt that integration increased coverage of HIV testing, particularly among difficult-to-reach populations, and improved convenience, efficiency, and confidentiality for women while reducing stigma. Pregnant women believed that early detection of HIV protected their own health and that of their children. Stigma surrounding HIV was reported to 4lead some women to discontinue services or seek care through other access points in the health system. | 1^st^ |
| 45 | Kilembe, 2015 | Prospective cohort study | Couples, **South Africa**  n = 907 (couples) | CVCT invitation from a promoter. Couples attended a group session i.e. if more than one couple was available or went straight into pre-test counseling. Underwent HIV testing and given results and post-test counseled over 12 weekends at 5 pilot clinics | Testing | Of the 907 couples (1,814 individuals) that underwent CVCT, the prevalence of HIV was 41.8 % and the prevalence of HIV serodiscordance was 29.5 % (19.3 % M-F+, 10.3 % M + F-). 245 (27 %) of couples were concordant positive and 56.6 % of all couples had at least one partner who was HIV positive. Of all HIV positive persons, 35.4 % had HIV negative partner. | 1^st^ |
| 46 | Mdodo, 2014 | Prospective cohort study | ≥13 yrs, MSM, **USA**  n=1072 | Provided free, confidential rapid HIV testing in accordance with LHD policy and in collaboration with local HIV testing agencies. Counseling offered, confirmatory testing done by either Western blot or second rapid HIV test at the LHD facility and linked to care by LHD partners. | Testing: first time testers | Seventy-seven (7.2%) participants tested preliminarily HIV positive (i.e., had a positive reactive rapid HIV test), of whom 15 (19.5%) had never tested previously for HIV, 39 (50.6%) had a negative test 12 months before the event, and 23 (29.9%) had a negative test >12 months before the event. | 1^st^ |
| 47 | Papanna, 2012 | Cross-sectional | Integrated Counselling and Testing Centres (ICTCs), **India**  n = 13 | Structure and process evaluation was carried out at Udupi district between July-September 2010 at all the 13 ICTCs functioning under NACP III. Data was collected from 2 ICTCs located in a Medical college hospital, 2 centres at District general hospital, 2 centres at Taluk hospitals, 4 centres at CHC, and 3 centres at PHC | Testing: Attendance | 99% (4784) of the clients attending pretest counselling underwent HIV testing and 96.5% (4660) attended the post-test counselling. | 1^st^ |
| 48 | Hartney, 2013 | Descriptive | Sexual health commissioners, **England**  n = 40 | Expanded HIV testing programs commissioned by sexual health commissioners in a non-traditional setting. Data obtained via survey. | Testing services offered: Self-reported | 31% (11/35) of high-prevalence primary care trusts (PCTs) had commissioned routine testing of new registrants in general practice. 14% (5/35) had commissioned routine testing of general medical admissions in a hospital covering their area. Community testing had been commissioned in 51% (18/35) of high-prevalence PCTs, taking place via outreach programmes carried out by charities and voluntary sector organizations. | 1^st^ |
|  |  |  |  |  | Feasibility: Response rate | 35/40 responded. / Highly feasible. | Other |
| 49 | Haskew, 2015 | Prospective cohort study | ≥15 yrs, HIV+, **Kenya**  n = 1752 | The Kenyan Ministry of Health and partners launched a community integrated prevention campaign (IPC). IPC data were compared to voluntary counselling and testing (VCT) data for 2Y period. | ART adherence: CD4 or VL | Patients testing HIV positive during the IPC had more than two times higher odds of presenting early with CD4 count greater than 350 cells/μl (adjusted OR 2.15, 95% CI 1.28 – 3.61, p = 0.004) and presenting early with WHO clinical stage 1 or 2 of HIV infection (adjusted OR 2.39, 95% CI 1.24 – 4.60, p = 0.01) at initial clinic visit compared to individuals who tested HIV positive via VCT services. | 3^rd^ |
| 50 | Madiba, 2015 | Feasibility study | 14-27 yrs, **South Africa** n = 2970 | Acceptability of HIV testing and counselling in schools. | Acceptability: Self-reported | The acceptability of HTC at school was high (n = 2282, 76.9%) and 2129 (71.8%) were willing to be tested at school. / Highly acceptable. | 1^st^ |
| 51 | Yan, 2014 | Cross-sectional | MSM, **China** | A "cash on service delivery" where community based organizations (CBOs) were paid various amounts per MSM test, diagnosed and linked to care. 10USD/MSM tested, 82USD /newly diagnosed HIV case and 50USD/PLHA receiving a defined package of followup care services. Task shifting to CBOs started in 2008. | ATT testing: Attendance rate | HIV testing increased from 4.1% in 2008 to 22.7% in 2012 | 1^st^ |
|  |  |  |  |  | ART adherence: CD4 | Coverage of CD4 tests increased from 71.1% in 2008 to 86.0% in 2012. Baseline median CD4 count of newly diagnosed HIV cases increased from 309cells/uL to 397cells/uL in 2012. | 3^rd^ |
| 52 | Pertersen, 2016 | Cross-sectional | **Kenya and Uganda**  n = 77773 | SEARCH study uses “test and treat” strategy in the communities of Kenya and Uganda. Interim results reported here. | Viral Suppression: VL | 89.5% were suppressed (95%CI:88.6%,90.4%). Population viral suppression at year 2 was 81.3% (95%CI: 80.3%,82.3%) . | 3^rd^ |
|  |  |  |  |  | Linked to care: on ART | 93.2% had received ART (95%CI:92.6%,93.9% . | 2^nd^ |
| 53 | MacKellar, 2016 | Cross-sectional | HIV+, **Tanzania**  n = 56907 | Bukoba Tanzania Combination Prevention Evaluation (BCPE) includes PITC, community based HTC (CBHTC) and integrated linkage case management. | Testing | 86% newly HIV diagnosed | 1^st^ |
|  |  |  |  |  | Linked to care: ART initiation | 90% registered for HIV care, of which 50% were initiated on ART | 2^nd^ |
| 54 | Fatti, 2016 | Cross-sectional | 10-19yrs, **South Africa**  n = 4800 | Combination intervention to increase HIV testing among adolescents. Testing interventions include; index client trailing, door-to-door testing, campaign testing. | ATT testing: first-time testers | 4756/4800 (99%) consented to HIV testing. Of this 90% males and 85.7% females were first time testers. | 1^st^ |
|  |  |  |  |  | Feasibility: participation rate | 54% tested in index client households, 40% tested during door-to-door visits and 6% tested at campaigns. | Other |
| 55 | Wamalwa, 2009 | RCT | 15 months to 12 years, HIV+, **Kenya**  n = 99 (intervention) n = 16 (control) | Medication diaries as the intervention, and standardized counselling without diary use as the control. | ART adherence: CD4 | Mean CD4 percentage was 17.2% in the diary arm versus 16.3% in the control arm at six months (p = 0.92), and 17.6% versus 18.9% at 15 months (p = 0.36). / No impact | 3^rd^ |
| 56 | Creek, 2007 | Prospective cohort | Pregnant women, **Botswana**  n=967 | Clinic workers provided group education and recommend HIV testing as part of routine antenatal care services. | Testing | The percentage of all HIV-positive women who knew their status at the time of delivery increased from 47% to 78%. The routine use of on-site rapid HIV tests in ANC clinics ensured that nearly all tested women received their results. | 1^st^ |
|  |  |  |  |  | Linked to care: on ART | The percentage receiving AZT increased from 29% to 56% from 2003 to 2004, The routine use of on-site rapid HIV tests in ANC clinics resulted was associated with 75% of women receiving AZT in 2005 and increasing numbers of women receiving NVP and ARV therapy. | 2^nd^ |
| 57 | Cantrell, 2008 | Cohort study | Food insecure adults, HIV+, on ART, **Zambia**  n=442 (Int) n=194 (Ctrl) | A food supplementation program alongside ART service expansion. Food insecure patients received monthly rations and ART adherence was measured during the first 12 months using the medication possession ratio (MPR). | ART adherence: pill count | Adherence to ART was higher among patients in the food group compared to controls. 258 of 366 (70%) of patients in the food group achieved an MPR of 95% or greater versus 79 of 166 (48%) among controls (RR=1.5; 95% CI: 1.2, 1.7) / Effective. | 3^rd^ |
| 58 | Kunutsor, 2012 | Cross-sectional | HIV+, ≥18 yrs, ART at least 3M, **Uganda**  n=856 | A combination of elements such as counseling, group education, leaflets, late attendee tracing, and adherence diaries was implemented. | ART adherence: pill count | Mean adherence (95% CI) improved statistically significant from baseline following implementation of the interventions (97.4% [96.9-97.9%] to 99.1% [99.0-99.3%], P0.001). There was also a significant difference between proportions with optimal (<95%) and suboptimal adherence (<95%) pre- and post-intervention (7.0% difference, 95% CI: 4.6-9.4%, P<0.001)./ Effective | 3^rd^ |

Mobile testing services

| No. | Author | Study Design | Participants/Country | Intervention | Measure/Metric | Results (MD, mean difference; RR, risk ratio; IRR, incidence rate ratio; HR, hazard ratio; SD, standard deviation, 95% CI when presented; Y, years; M, months; W, weeks) | 90-90-90 Target |
| --- | --- | --- | --- | --- | --- | --- | --- |
| 59 | Bassett, 2013 | Prospective cohort study | ≥15yrs, **Durban, South Africa**  n = 4703 (Int - mobile unit) n = 2254 (Ctrl - Ithembalabantu “The People's Hope” Clinic (IPHC)) | IPHC offers community-based mobile testing and testing at clinic based testing. 1-2 mobile units deployed/day to nearby community areas. Pre and post-test counselling offered. | Testing: follow-up appointment | Mobile testers were less likely to undergo CD4 testing (33% vs. 83%, respectively, p<0.001), but they have higher CD4 counts [median (interquartile range) 416 (287–587) cells/μL *vs.* 285 (136–482) cells/μL, respectively] than clinic testers (both *P* < 0.001). CD4 test retrival done at clinic. / Effective | 1^st^ |
|  |  |  |  |  | Testing | 4703 Mobile testers vs. 2254 clinic testers. | 1^st^ |
|  |  |  |  |  | Linked to care | Of those who tested HIV positive, 10% of mobile testers linked to care, vs. 72% of clinic testers (P < 0.001). | 2^nd^ |
| 60 | Milligan, 2014 | Descriptive | Students from **North Carolina** Private University or Community College aged 13-65, unknown HIV status  n=1408 (n=1000 private university, n=408 community college) | Testing sites at high traffic areas on campus Oral swab test was collected and results were given within 20 minutes. Intervention was run by students from the schools. All tested students received t-shirts | ATT testing: first-time testers | 56% were tested for the first time. |  |
| 61 | Fernández-Balbuena, 2014 | Cross-sectional | **Spain**  n=7552 | Mobile van offering free rapid HIV testing and brief counselling. | Testing: first-time testers | 47% of participants were first-time testers. Prevalence of HIV infection in this group was 0.6% (0.1% for both heterosexual men and women, and 3% MSM). | 1^st^ |
|  |  |  |  |  | Acceptability: Self-reported | 62% said that they had undergone the test only because they had passed by the van. | Other |
| 62 | September, 2016 | Cross-sectional | **South Africa**  n = 3921 | Desmond Tutu TB Centre implemented 1) Mobile HIV counselling and testing services (HCT) using tents and van at major city transport hub 2) Door-to-door HCT targeting people in high HIV/TB areas to increase testing. | Testing | 3859 / 3921 (98%) received HIV testing. | 1^st^ |
|  |  |  |  |  | Linked to care:: Self reported | Linkage to care was higher in door-to-door mobilisation (61% males, 58% females) compared to transport hub HCT (32% males, 44% females). | 2^nd^ |
| 63 | Ngassa, 2016 | Cross-sectional | **South Africa**  n = 4557 | The Mobile Clinic Program utilised 2 vans dispatched to highly populated areas in Johannesburg to increase HIV testing. | Testing: attendance | 4557 accessed the clinic, 94% were offered HCT, 7% were HIV+ | 1^st^ |
|  |  |  |  |  | Testing: first time testers | 39% were first time testers or had tested more than 12M prior. | 1^st^ |
| 64 | Chamie, 2015 | Cross-sectional | ≥15yrs, **Uganda and Kenya**  n = 168336 | 2W mobile multi-disease community health campaign followed by home-based testing | Testing | 134697 / 168336 (80%) tested for HIV. HIV prevalence was 9.4% | 1^st^ |
|  |  |  |  |  | Testing: first time testers | 43% of adults tested were first time testers. | 1^st^ |
| 65 | Larson, 2012 | Pilot Study | >18 yrs, **South Africa**  n = 311  (intervention)  n = 197 (control) | A mobile HIV counseling and testing (HCT) program around Johannesburg piloted the integration of point- of-care (POC) CD4 testing, using the Pima analyzer, to improve linkages to HIV care. Intervention group received the POC CD4 and control was not. | Linked to care: Self-reported | No differences in patient characteristics were observed between the 2 groups. Approximately 62.7% of patients were successfully followed up 8 weeks after HIV testing, with no differences observed between testing groups. Among those followed up, 54.4% reported completing their referral visit. Patients offered the POC CD4 test were more likely to complete the referral visit for further HIV care (RR 1.25, 95% confidence interval: 1.00 to 1.57). | 2^nd^ |

Campaigns

| No. | Author | Study Design | Participants/Country | Intervention | Measure/Metric | Results (MD, mean difference; RR, risk ratio; IRR, incidence rate ratio; HR, hazard ratio; SD, standard deviation, 95% CI when presented; Y, years; M, months; W, weeks) | 90-90-90 Target |
| --- | --- | --- | --- | --- | --- | --- | --- |
| 66 | Fernandez-Balbuena, 2015 | Quasi-experimental | **Spain**  n = 3251 | Me´dicos del Mundo (MdM) offers free HIV testing and counseling oriented to vulnerable populations in a non-clinical setting. MdM has been performing traditional HIV tests since 2003 and rapid testing since 2007. | Testing: first time testers | 72 new diagnoses between 2008-2012. 1027/3251 were first-time testers. | 1^st^ |
|  |  |  |  |  | Testing | Compared to 2 surveillance systems, EPIVIH and SNHSS, MdM was able to reach more female sex workers (FSW). p = 0.0023 MdM vs. EPIVIH, p < 0.0001 MdM vs. SNHSS. / Effective | 1^st^ |
| 67 | Boyer, 2014 | Cross-sectional | 13-24 yrs old, HIV risk, Hispanic/Latino, USA and Puerto Rico  n = 1595 | Comparison of HIV testing outreach programs: an alternative venue-based testing (AVT) strategy and a social and sexual network referral (SSNIT) strategy to improve HIV testing. | Testing | 784 and 812 participants recruited through AVT and SSNIT respectively. 3/812 (0.37%) recruited via SSNIT and 4/784 (0.51%) via AVT were HIV infected. All received post-test counselling. | 1^st^ |
|  |  |  |  |  | Linked to care | 1/3 in SSNIT and 3/4 in AVT were successfully linked to care | 2^nd^ |
| 68 | Limaye, 2016 | Feasibility study | **Malawi**  n = 4200 text messages | Impact of Chenicheni Nchiti? (‘What is Real?’), a 30-minute weekly national radio program addressing HIV/AIDS related issues. Influence of the show was determined via feedback texts received from listeners over 2 3M periods, texts chosen at random. | Acceptability: Self-reported | Listeners reported that the program helped them realize the importance of HIV testing, provided courage and motivation to get tested. | 1^st^ |
| 69 | Marlin, 2014 | Feasibility study | ≥18 yrs, MSM, African American, **USA**  First testing period n=237, second testing period n=404 | A commercial voucher program for free OraQuick® In-Home HIV Test kits targeting high-risk African American MSM in LA | Feasibility | Distributors confirmed that 62 of 237 (26.2%) vouchers supplied during the first test period in July 2013 were distributed. 10 (16.1%) of 62 distributed vouchers were redeemed. During the second test period from August through December 2013, 230 of 404 (56.9%) vouchers were distributed. Forty-three (18.7%) of the distributed vouchers were redeemed. / Low feasibility | 1^st^ |
| 70 | Silvestre, 2016 | Cross-sectional | 15-49yrs, **Swaziland**  n = 845 | Exposure to OneLove campaign aiming to bring about behaviour changes and condom use via various forms of media (e.g tv, radio, books, posters). Study focuses on mobile populations. | Testing: Self-reported | Significant difference in HIV testing in those exposed to campaign in Swaziland and neighbouring country vs those not exposed (AOR=2.9, CI 1.1-8.0. Significant difference in HIV testing in men (OR=3.6, 95%CI 1.4-9.4). / Effective | 1^st^ |
| 71 | Van Handel, 2014 | Quasi-experimental | **USA**  n = 63914 (int) n = 48748 (cntrl) n = 49 182 (cntrl) | Determine if National HIV Testing Day increased HIV testing and diagnosis. | Testing | On average 15,000 more tests were conducted during NHTD vs the control weeks (p<0.0001). 467 newly identified HIV+ cases were found during NHTD compared to control week 1 and 2, 367 (p<0.0005) and 356 respectively (p<0.0001)./ Effective | 1^st^ |

Technology (SMS/alarms/reminders, etc)

| No. | Author | Study Design | Participants/Country | Intervention | Measure/Metric | Results (MD, mean difference; RR, risk ratio; IRR, incidence rate ratio; HR, hazard ratio; SD, standard deviation, 95% CI when presented; Y, years; M, months; W, weeks) | 90-90-90 Target |
| --- | --- | --- | --- | --- | --- | --- | --- |
| 72 | Mushamiri, 2015 | Retrospective cohort study | Pregnant, **Kenya**  n = 650 | The Millennium Villages Project (MVP) implemented in Western Kenya a mobile Health tool that uses text messages to coordinate Community Health Worker (CHW) activities around antenatal care (ANC) and Prevention of Mother-to-Child Transmission of HIV (PMTCT), named the ANC/PMTCT Adherence System (APAS) | Testing: follow-up appointment | 348/650 women made their 1st ANC visit in the 2nd trimester of their pregnancy. The HIV prevalence in the investigated population was 27.1%, with 176 being HIV+ and 474 being HIV-. | 1^st^ |
| 73 | Gupta, 2016 | Feasibility | HIV-exposed babies, **India**  n=1139 (pre-intervention) n=1631 (post-intervention) | Early Infant Diagnosis Follow-up System (EID Follow-up system) sends automated reminders to field staff to follow-up with HIV-exposed babies. Reminders sent 7 days prior to follow-up date. | Testing: follow-up | Prior to EID Follow-up System introduction 55.9% (637/1139) of the HIV exposed babies born were tested at 6 weeks for DNA-Polymerase Chain Reaction during April 2011– March 2012. After its introduction, 68.4% (1117/1631) of them were tested during April 2012–March 2013. Correspondingly, the 18 months confirmatory HIV testing in eligible babies increased from 45.6% (934/2044) to 54.7%(1118/2044) during the same period. | 1^st^ |
| 74 | Chang, 2011 | RCT (Cluster-randomized trial) | HIV+, **Uganda**  n=446 (Int)  n=524 (Ctrl) | An mHealth (mobile phone) support intervention used by community-based peer health workers (PHW) on AIDS care in rural Uganda. | ART adherence: VL | The primary outcome of any virologic failure was not significantly different between study arms (19.4% vs. 16.4%, mHealth+ vs. mHealth− Arm respectively). / No impact | 3^rd^ |
| 75 | Chung, 2011 | RCT | HIV+, ≥18 yrs, ART naïve, **Kenya**  n=200 (counseling) n=200 (alarm device) | Participants were exposed either to counseling or an alarm device to assist with ART adherence. | ART adherence: VL CD4 | Adherence during the first month after initiating HAART was significantly higher among those who received counseling (difference in intercepts, 3.58%; 95% CI 0.50%–6.66%; p = 0.023), and this difference was constant over 18 mo follow-up (difference in slopes, 0.13% per month; 95% CI −0.16% to 0.42%; p = 0.4). No significant differences were found in adherence <95% between those who received counseling and those who did not (HR 0.89; 95% CI 0.70–1.12; p = 0.3). | 3^rd^ |
| 76 | Dowshen, 2012 | Prospective pilot cohort | HIV+, 14-29 yrs, use a cell phone, on ART, **USA**  n=25 | Participants received personalized daily SMS reminders and a follow-up message 1 hour later assessing whether they took the medication, and asking participants to respond via text message. | ART adherence: self-reported, CD4, VL | Mean visual analogue scale scores significantly increased at 12 and 24 weeks in comparison with baseline (week 0: 74.7, week 12: 93.3, P < .001; week 24: 93.1, P < .001). There was no significant difference in CD4 cell count or viral load between baseline and 12- or 24-week follow-up. | 3^rd^ |
| 77 | Hardy, 2011 | RCT | HIV+, low ART adherence (<85%), **USA**  n=12 (CP group)  n=11 (BP group) | Participants were randomized to either a cellular phone (CP) or beeper (BP). CP subjects received personalized text messages daily; in contrast, BP subjects received a reminder beep at the time of dosing. Interviews were scheduled at weeks 3 and 6. | ART adherence: self –reported & pill count | Adherence measured by pill count and self-reported increased relative to baseline to 69.1% ± 22.6 and 85.3% ± 19.2, respectively. | 3^rd^ |
|  |  |  |  |  | ART adherence: MEMs and CAS | The MEMS and CAS adherence was estimated to be 72.0% ± 26.4 and 69.7% ± 24.1, respectively. Significant differences between the mean adherence of the two intervention groups (Aremind-CP or BP) were found when adherence was measured by MEMS (mean difference ± SD: 28.1 ± 10.0, p = 0.012) and CAS (24.8 ± 9.4, p = 0.018)./ Effective | 3^rd^ |
| 78 | Simoni, 2011 | RCT | HIV+, ≥18 yrs, ART eligible, **China**  n=36 (Int) n=34 (Ctrl) | Participants assigned to the enhanced intervention could choose an alarm device, counseling (three sessions of up to 1 hour each), or both. Participants in the minimal arm were the control and received no further adherence prompts | ART adherence: self-reported | 100% adherence in the 30 days prior to the assessment decreased over the course of the study (from 94 to 86%) in the minimal intervention arm. 100% adherence in the enhanced intervention increased from 85% to 94%. At the 19-week assessment (100% vs. 86%), χ2 (1) = 5.05, P = 0.04 / Effective | 3^rd^ |
|  |  |  |  |  | ART adherence: EDM (electronic drug monitoring) | Participants in the enhanced intervention had greater average 30-day dose (83% vs. 76%) and timing (71% vs. 59%) adherence than those in the minimal intervention by the 7-week assessment and they continued to have better adherence until the final follow-up assessment. / Effective | 3^rd^ |
| 79 | Pop-Eleches, 2011 | RCT | HIV+, ≥18 yrs, ART within 3 months, **Kenya**  n=139 (Ctrl) n=70 (short daily reminders) n=72 (long daily reminders) n=73 (short weekly reminders) n=74 (long weekly reminders) | Participants received SMS reminders that were either short or long and sent at a daily or weekly frequency. Participants were expected to return to the clinic once a month. | ART adherence: pill count | 53% of participants receiving weekly SMS reminders achieved adherence of at least 90% during the 48wks of the study, compared with 40% of participants in the control group (P=0.03). Participants in groups receiving weekly reminders were also significantly less likely to experience treatment interruptions exceeding 48 h during the 48-week follow-up period than participants in the control group (81 vs. 90%, P = 0.03). / Effective | 3rd |
| 80 | Sabin, 2010 | RCT | HIV+, ≥18 yrs, on ART, **China**  n=35 "low" adheres (n=18 Int, n=17 Ctrl) n=33 "high" adheres (n=16 Int, n=17 Ctrl) | A study team member downloaded and reviewed the subject’s previous month’s electronic drug monitor (EDM) data monthly. Each subject found to be less than 95% adherent according to the EDM data was ‘flagged’ for counseling with a clinic physician or nurse utilizing the EDM information immediately following regular clinic visit activities. | ART adherence: CD4 | Mean adherence and CD4 count were 86.8 vs. 83.8% and 297 vs. 357 cells/μl in intervention vs. control subjects. At month 12, among 64 subjects who completed the trial, mean adherence had risen significantly among intervention subjects to 96.5% but remained unchanged in controls. Mean CD4 count rose by 90 cells/μl and declined by 9 cells/μl among intervention and control subjects, respectively. | 3^rd^ |
| 81 | Lester, 2010 | RCT | HIV+, ≥18 yrs, ART naive, access to mobile phone, **Kenya**  n=273 (Int) n=265 (Ctrl) | Patients received weekly SMS messages from a clinic nurse and were required to respond within 48 hours. Control patients received no text messages. | ART adherence: self-reported | Self-reported adherence remained significantly better in the SMS group than the control group (odds ratio [OR] 0·57, 95% CI 0·40–0·83; p=0·0028)./ Effective | 3^rd^ |
|  |  |  |  |  | ART adherence: VL | Weak evidence of improved suppression of viral load in the SMS group compared with the control group (OR 0·71, 95% CI 0·50–1·01; p=0·058). / No impact | 3^rd^ |
| 82 | Mbuagbaw, 2012 | RCT | HIV+, ≥21 yrs, on ART, access to mobile phone, **Cameroon**  n=101 (Int) n=99 (Ctrl) | Patients received short, weekly SMS messages with altering motivational messages also reminding them to take their medication. Messages made no mention of HIV. Control patients received no text messages. | ART adherence: self-reported | (risk ratio [RR] 1.06, 95% confidence interval [CI] 0.89, 1.29; p = 0.542; reported missed doses (RR 1.01, 95% CI 0.87, 1.16; p>0.999) / No impact | 3^rd^ |
|  |  |  |  |  | ART adherence: pharmacy refills | MD=0.1, 95% CI: 0.23, 0.43; p = 0.617. / No impact | 3^rd^ |
